# Dual Atrioventricular Nodal Physiology in Sinus Rhythm: An Extraordinary Manifestation of a Common Substrate

**DOI:** 10.1016/j.jaccas.2026.108493

**Published:** 2026-07-29

**Authors:** Ignacio Pascual Ramos, Isabel López Alacid, Ez Alddin Rajjoub Al-Mahdi, Celia Denche Sanz, Daniel Rodríguez Muñoz

**Affiliations:** Department of Cardiology, Hospital Universitario 12 de Octubre, Madrid, Spain

**Keywords:** ablation, electrocardiogram, electrophysiology

## Abstract

**Case Summary:**

A 32-year-old woman was evaluated for experiencing episodes of palpitations, which were never documented. Complementary test results were normal. A 12-lead Holter monitor was requested, and a manifestation of dual nodal physiology was observed. This was key to indicate an electrophysiological study, as this increased pretest likelihood of atrioventricular nodal re-entry tachycardia. In the electrophysiological study, we identified 2 families of atrio-hisian intervals of 106 milliseconds and 304 milliseconds, respectively, with an his-ventricular interval of 52 milliseconds. With atrial programmed stimulation, a nodal jump was evidenced, and a regular, narrow QRS tachycardia with atrioventricular relation of 1:1 and a ventriculo-atrial interval of 19 milliseconds was induced, which is compatible with atrioventricular nodal reentry tachycardia, so slow pathway ablation was performed.

**Take-Home Message:**

Manifestations of dual nodal pathways are uncommon in sinus rhythm, but they should be recognized, as they demonstrate a potential arrhythmic substrate in patients with palpitations.

## Visual Summary

**Figure:**
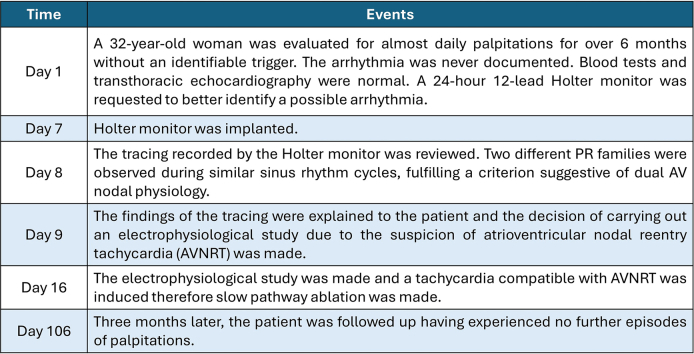

**Visual Summary:**
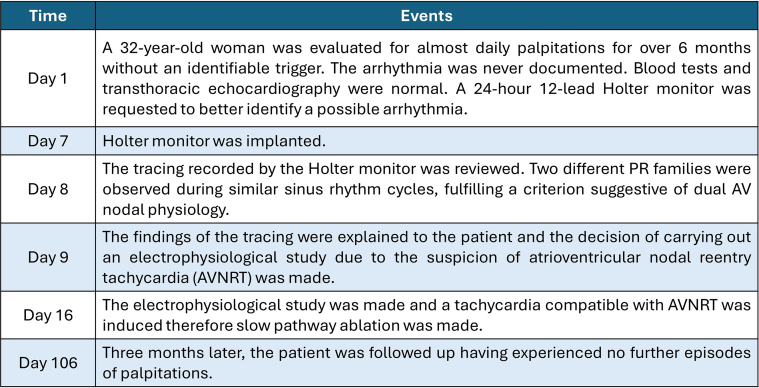
Timeline of the Case

## Case Presentation

A 32-year-old woman was evaluated for almost daily palpitations for over 6 months without an identifiable trigger. The arrhythmia was never documented because the tachycardia had resolved once the electrocardiogram was recorded. She had no family history of cardiac disease nor was receiving chronic treatment. Blood test and transthoracic echocardiography results were normal. A 24-hour 12-lead Holter monitor was requested to better identify a possible arrhythmia, and this trace was observed (Figure 1).

**Take-Home Message**

• Manifestations of dual nodal pathways are uncommon in sinus rhythm, but they should be recognized, as they demonstrate a potential arrhythmic substrate in patients with palpitations.

**Figure 1:**
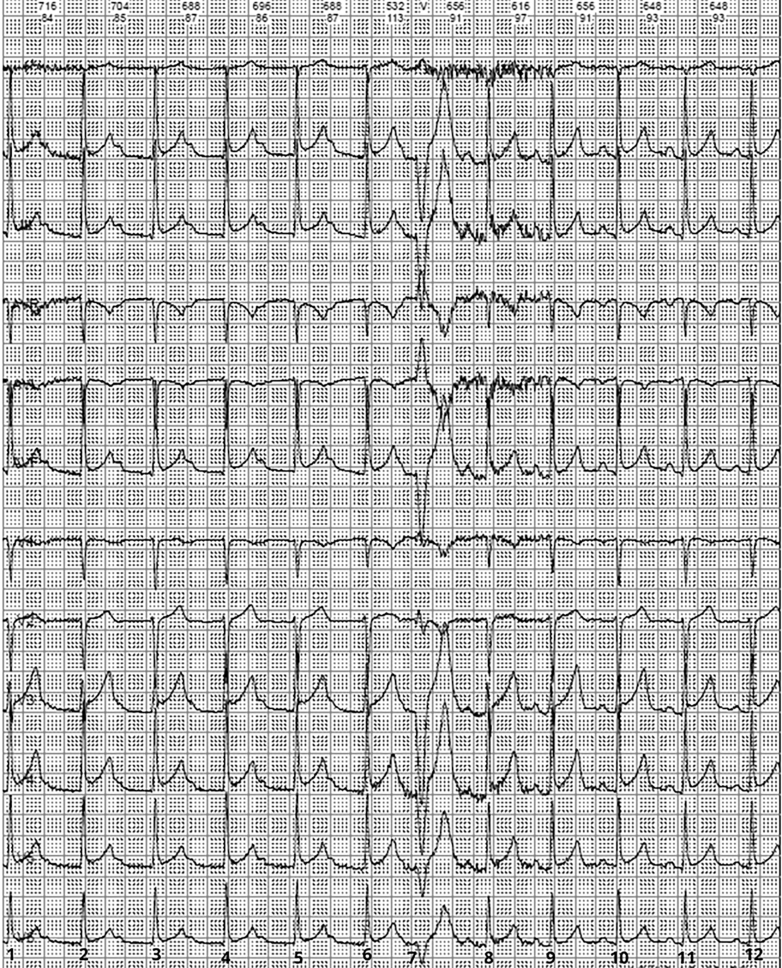
12-Lead Holter Trace Configuration was 10 mm/mV and 25 mm/s. See how the PR interval shortens abruptly after the ventricular extrasystole.

## Question

Regarding the phenomenon shown on the electrocardiogram:

A. The beat with wide QRS complex is highly likely conducted with pre-excitation through a conventional accessory pathway.
B. Manifestations of dual atrioventricular (AV) node physiology are easily identified during sinus rhythm.
C. The linking phenomenon explains the maintenance of a functional block in the fast pathway (FP).
D. An abrupt change in PR interval duration is the only manifestation of dual AV nodal physiology during sinus rhythm.

Correct answer: C.

Two different PR families are observed during similar sinus rhythm cycles, fulfilling a criterion suggestive but not definitive of dual AV nodal physiology. Key elements supporting conduction over different pathways include the abrupt PR interval change after a ventricular extrasystole (VE), as well as the presence of the “linking”^1^ (persistence of functional block in one pathway due to repeated concealed penetration by impulses conducted over another limb of the circuit) and “peeling-back”^1^ (premature concealed conduction that shortens effective refractoriness, facilitating subsequent conduction) phenomena (Supplemental Figure 1).

PR interval is initially prolonged. After a VE, subsequent sinus beats show a shorter PR interval. During beats 1-6, anterograde conduction occurs via the slow pathway because the FP is blocked by concealed retrograde conduction.^2^

No electrocardiographic evidence of retrograde atrial activation is observed. This is explained by concealed retrograde conduction into the FP that fails to reach the atrium and gets blocked but leaves the FP refractory, preventing subsequent anterograde conduction (linking).^1^ Alternatively, an interference-type linking mechanism may occur, with alternating concealed anterograde and retrograde wave fronts interacting at a critical site in the FP, maintaining local refractoriness and block.

The seventh beat is a VE, after which FP conduction resumes (beats 8-12), confirming that linking maintained the FP block. Recovery of FP conduction after the VE may be explained by 2 mechanisms. First, premature concealed conduction through the FP may allow earlier termination of its refractory period, before the next sinus impulse arrives (peeling-back).^1^ Second, narrowing of the refractory window due to earlier tissue depolarization (cycle-length–dependent shortening of refractoriness) may occur. However, this mechanism is typically observed in the atria, ventricles, and the His-Purkinje system, rather than in the AV junction.

Answer A is incorrect because the prolonged PR interval argues against pre-excitation through a conventional accessory pathway. Manifestations of dual AV nodal physiology are uncommon during sinus rhythm, although 2 PR families and PR alternans are recognized but rare manifestations of dual AV nodal physiology,^3^ as are dual ventricular responses to a single supraventricular impulse. According to this, answers B and D are incorrect.

## Funding Support and Author Disclosures

The authors have reported that they have no relationships relevant to the contents of this paper to disclose.

## Appendix

Supplemental Figure 1 Ladder Diagram Leads I, II, III, and aVR are shown. In the first 6 beats, conduction through the fast pathway is blocked due to retrograde concealed conduction. After the ventricular extrasystole, conduction through the fast pathway is recovered.
