# Supplementary material for: Dual Atrioventricular Nodal Physiology in Sinus Rhythm: An Extraordinary Manifestation of a Common Substrate
Source: JACC Case Rep. 2026 Jul 29;31(30):108493. doi: 10.1016/j.jaccas.2026.108493 (PMC13420554; doi:10.1016/j.jaccas.2026.108493)
Supplement: Supplemental Figure 1 — Ladder Diagram Leads I, II, III, and aVR are shown. In the first 6 beats, conduction through the fast pathway is blocked due to retrograde concealed conduction. After the ventricular extrasystole, conduction through the fast pathway is recovered. [file mmc1.docx]

Supplemental Appendix


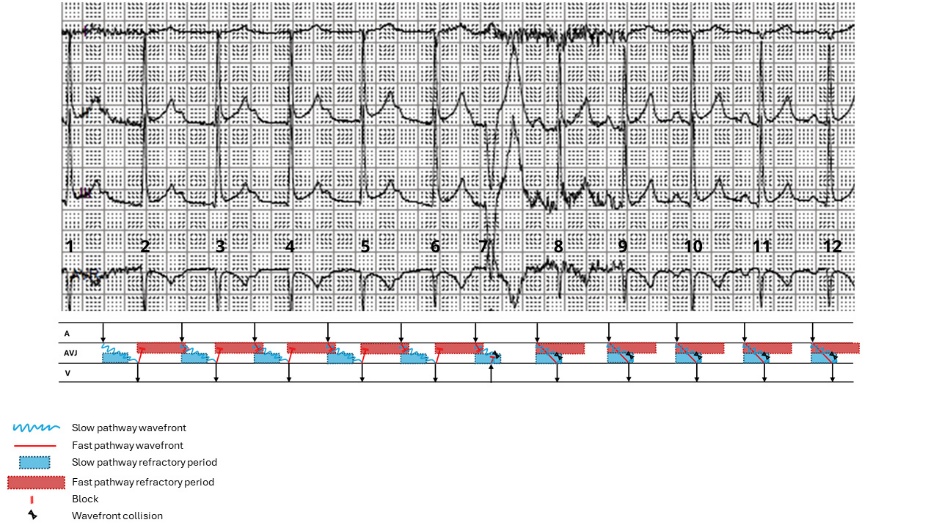


**Supplemental Figure 1.** **Ladder diagram**. Leads I, II, III and aVR are shown. In the six first beats, conduction through fast pathway is blocked due to retrograde concealed conduction. After the ventricular extrasystole, conduction through fast pathway is recovered.
